# Psychometric comparison of three behavioral scales for the assessment of pain in critically ill patients unable to self-report

**DOI:** 10.1186/cc14569

**Published:** 2015-03-16

**Authors:** G Papakitsos, A Kapsali, T Papakitsou

**Affiliations:** 1 GHA, Arta, Greece; 2 General Hospital Messologi, Greece

## Introduction

Pain assessment is associated with important outomes in ICU patients but remains challenging, particularly in noncommunicative patients. Use of a reliable tool is paramount to allow any implementation of sedation/analgesia protocols in a multidisciplinary team. This study compared psychometric properties (inter-rater agreement primarily; validity, responsiveness and feasibility secondarily) of three pain scales: Behavioural Pain Scale (BPS/BPS-NI, that is BPS for non-intubated patients), Critical Care Pain Observation Tool (CPOT) and Non-Verbal Pain Scale (NVPS), the pain tool routinely used in this 16-bed medical ICU.

## Methods

In a prospective observational study of ED polytraumatized patients (*n *= 23, mean Acute Physiology and Chronic Health Evaluation II (APACHE II) score of 11 ± 6) we measured (in the first 24 hours) plasma TAC by the ferric reducing activity/antioxidant power (FRAP). For control subjects, we used age-matched and gender-matched volunteers (*n *= 32). We also evaluated the contribution of antioxidant molecules (uric acid, bilirubin, and albumin) to these values.

## Results

Polytraumatized patients show differences in TAC with reference to control subjects. ED polytraumatized patients show high FRAP values. We found that FRAP values were inversely correlated with APACHE II score (*r *= -0.266, *P *< 0.01) suggesting that, in trauma patients, increased antioxidant response, as measured by the FRAP assay, could be a pathophysiological response to stress. Albumin and uric acid concentrations reproduced the FRAP trend with severity.

## Conclusion

FRAP values in trauma ED patients are independently influenced by age (β = 0.271, *P *< 0.021), APACHE II score (β = -0.356, *P *< 0.002) and head trauma (β = -0.219, *P *< 0.045). These results accentuate the influence of trauma location and severity in TAC changes. The TAC response in ED patients reinforces the need for adequate tailoring of treatments aimed at their recovery, such as antioxidant therapies.

